# Long-Term Survival Following Surgical Ablation for Atrial Fibrillation Concomitant to Isolated and Combined Coronary Artery Bypass Surgery—Analysis from the Polish National Registry of Cardiac Surgery Procedures (KROK)

**DOI:** 10.3390/jcm9051345

**Published:** 2020-05-04

**Authors:** Mariusz Kowalewski, Marek Jasiński, Jakub Staromłyński, Marian Zembala, Kazimierz Widenka, Michał Oskar Zembala, Krzysztof Bartuś, Tomasz Hirnle, Inga Dziembowska, Piotr Knapik, Marek Deja, Waldemar Wierzba, Zdzisław Tobota, Bohdan J. Maruszewski, Piotr Suwalski

**Affiliations:** 1 Clinical Department of Cardiac Surgery, Central Clinical Hospital of the Ministry of Interior and Administration, Centre of Postgraduate Medical Education, 02507 Warsaw, Poland; jakubstaromlynski@gmail.com (J.S.); suwalski.piotr@gmail.com (P.S.); 2 Department of Cardio-Thoracic Surgery, Heart and Vascular Centre, Maastricht University Medical Centre, 6202 AZ Maastricht, The Netherlands; 3 Thoracic Research Centre, Nicolaus Copernicus University, Collegium Medicum in Bydgoszcz, Innovative Medical Forum, 85796 Bydgoszcz, Poland; 4 Department and Clinic of Cardiac Surgery, Wroclaw Medical University, 50367 Wroclaw, Poland; marekjasinski@yahoo.pl; 5 Department of Cardiac, Vascular and Endovascular Surgery and Transplantology, School of Medicine with the Division of Dentistry in Zabrze, Medical University of Silesia, 40055 Katowice, Poland; m.zembala@sccs.pl (M.Z.); m.zembala.jr@sccs.pl (M.O.Z.); 6 Division of Cardiac Surgery, Heart and Lung Transplantation and Mechanical Circulatory Support, Silesian Center for Heart Disease, 41800 Zabrze, Poland;; 7 Clinical Department of Cardiac Surgery, District Hospital no. 2, University of Rzeszów, 35301 Rzeszów, Poland; kazw@poczta.onet.pl; 8 Department of Cardiovascular Surgery and Transplantology, Jagiellonian University Medical College, John Paul II Hospital, 31202 Krakow, Poland; krzysztofbartus@gmail.com; 9 Department of Cardiosurgery, Medical University of Bialystok, 15276 Bialystok, Poland; hirnlet@wp.pl; 10 Department of Pathophysiology, Faculty of Pharmacy, Collegium Medicum, Nicolaus Copernicus University, 87100 Toruń, Poland; i.dziembowska@gmail.com; 11 Department of Anaesthesiology, Intensive Therapy and Emergency Medicine, Silesian Centre for Heart Diseases, Medical University of Silesia, 41800 Zabrze, Poland; p.knapik@sccs.pl; 12 Department of Cardiac Surgery, School of Medicine in Katowice, Medical University of Silesia, 40055 Katowice, Poland; mdeja@sum.edu.pl; 13 Department of Cardiac Surgery, Upper-Silesian Heart Center, 40635 Katowice, Poland; 14 Satellite Campus in Warsaw, University of Humanities and Economics in Łódź, 01513 Warsaw, Poland; wwierzba@post.pl; 15 Department of Pediatric Cardiothoracic Surgery, The Children’s Memorial Health Institute, 04730 Warsaw, Poland; zdzislaw.tobota@magnum2.pl (Z.T.); bmar@ecdb.pl.pl (B.J.M.)

**Keywords:** Coronary artery bypass grafting, registry, concomitant surgical ablation, long-term results, atrial fibrillation

## Abstract

The current investigation aimed to evaluate long-term survival in patients undergoing isolated and combined coronary artery bypass grafting (CABG) with concomitant surgical ablation for atrial fibrillation (AF). Procedural data from KROK (Polish National Registry of Cardiac Surgery Procedures) were retrospectively collected. Eleven thousand three hundred sixteen patients with baseline AF (72.4% men, mean age 69.6 ± 7.9) undergoing isolated and combined CABG surgery between 2006–2019 in 37 reference centers across Poland and included in the registry were analyzed. The median follow-up was four years (3.7 IQR 1.3–6.8). Over a 12-year study period, there was a significant survival benefit (Hazard Ratio (HR) 0.83; (95% Confidence Interval (CI): 0.73–0.95); *p* = 0.005) with concomitant ablation as compared to no concomitant ablation. After rigorous propensity matching (LOGIT model, 432 pairs), concomitant surgical ablation was associated with over 25% improved survival in the overall analysis: HR 0.74; (95% CIs: 0.56–0.98); *p* = 0.036. The benefit of concomitant ablation was maintained in the subgroups, yet the most benefit was appraised in low-risk patients (EuroSCORE < 2, *p* = 0.003) with the three-vessel disease (*p* < 0.001) and without other comorbidities. Ablation was further associated with significantly improved survival in patients undergoing CABG with mitral valve surgery (HR 0.62; (95% CIs: 0.52–0.74); *p* < 0.001) and in patients in whom complete revascularization was not achieved: HR 0.43; (95% CIs: 0.24–0.79); *p* = 0.006.

## 1. Introduction

Advancements in the understanding of the pathogenesis of atrial fibrillation (AF) and the development of new technologies have resulted in numerous improvements in the surgical ablation of AF, particularly in patients with mitral valve (MV) disease. The above was partially reflected in the 2017 Society of Thoracic Surgeons (STS) Clinical Practice Guidelines for the surgical treatment of AF assigning class IA recommendations for surgical ablation at the time of concomitant MV operations to restore sinus rhythm (SR) [1]. Similarly, class IB recommendation is assigned for surgical ablation at the time of isolated and combined coronary artery bypass grafting (CABG).

Unlike MV surgery with the prevalence of surgical ablation for AF reaching up to 70% and also increasing trends seen in the latest STS database report [2], concomitant ablation is performed in only approximately 30% of patients with AF undergoing CABG. In previous reports, in up to 60% of patients undergoing cardiac surgery, concomitant AF was left untreated. This may be due to the variety of lesion sets and energy sources, but more probably is due to the lack of clinically convincing remote results [3].

Left untreated, atrial fibrillation increases mortality and morbidity in patients undergoing cardiac surgery [4,5]. While the MAZE procedure effectively eliminates AF in most of these patients, its complexity and increased operative time have precluded its widespread application [6] and, in particular, during CABG.

In the current analysis, we report long-term survival results after concomitant surgical ablation for atrial fibrillation in patients undergoing isolated and combined CABG procedures from the KROK, Polish National Registry of Cardiac Surgery Procedures.

## 2. Experimental Section

### 2.1. Registry Design

Data were collected in a retrospective fashion from the KROK (Polish National Registry of Cardiac Surgery Procedures) registry (available at www.krok.csioz.gov.pl). The registry is an ongoing, nationwide, multi-institutional registry of all general heart surgery procedures in Poland, which collects in-hospital data on patients and outcomes; the details on registry design have been described before [7]. Long-term survival data are transferred to the registry directly from NHS records—registry modules collecting the data on surgical ablation types and strategies, as well as the history and status of AF were not available at the time of study conception.

### 2.2. Data Collection

A detailed questionnaire, defined according to standard definitions, including demographic data, history, physical findings, management, imaging studies, and outcomes, was developed. Anonymous patient data were collected either at presentation or by physician review of the hospital records and were forwarded to the KROK registry. The forms were reviewed for clinical face validity and internal analytical validity.

### 2.3. Study Population and Clinical Variables

Using the KROK participant user file, we identified adult patients undergoing CABG surgery between January 2006–December 2019. Of 193,488 CABG surgery records, we excluded those without a history of AF or AF at the time of presentation. Both isolated and combined CABG surgeries were included. Excluded were records in which the number of distal anastomoses and type of used graft material could not be determined. Additionally, we removed the records if the mode of surgery (e.g., off-pump (OPCAB), cardiopulmonary bypass (CPB)-CABG, aortic no-touch) were not available. No further exclusion criteria were imposed with regard to patients’ baseline status. For patients undergoing CABG surgery, we considered and reported three categories of variables as potentially influencing the primary endpoint: (1) baseline demographics: age, gender, EuroSCORE [8], diabetes, body mass index, hypertension, poor mobility, pulmonary hypertension, chronic kidney disease, vascular disease, chronic lung disease, and left ventricle ejection fraction (LVEF); (2) extent of coronary artery disease (CAD): previous myocardial infarction (MI), previous percutaneous coronary intervention (PCI), left main (LM) disease; and (3) surgical characteristics: redo, endocarditis, cardiogenic shock, intra-aortic balloon pump (IABP), critical preoperative state, intravenous (iv.) inotropes/nitrates, mode of surgery, total arterial revascularization (TAR)), completeness of revascularization, and concomitant procedures.

The primary endpoint assessed was long-term survival in concomitant ablation vs. no concomitant ablation. Analyses of early postoperative mortality (< 24 h) and 30-day mortality were performed as well. In-hospital complications, as well as the length of intensive care unit (ICU) and hospital stay (HLoS), were reported.

### 2.4. Statistical Analysis

Continuous, normally distributed variables were summarized as mean ± standard deviation; variables with non-normal distributions were summarized as median (interquartile range; IQR) and compared with the Mann–Whitney *U* test or standard *t*-test as appropriate. Categorical variables were expressed as number (%) and compared with the Fisher exact test. Cox proportional-hazards models were used to determine factors related the event-free survival. The ensuing statistical models were used to define the Hazard Ratios (HRs) point estimate and 95% confidence intervals (95% CI) of the effect size and to evaluate the efficacy of ablation with respect to CABG surgery. Respective HRs for the comparison of concomitant ablation vs. no concomitant ablation were calculated and reported first for the univariate Cox proportional-hazards model, taking into account all sets of variables categorized by (1) baseline demographics; (2) extent of CAD, and (3) surgical characteristics. Next, a multivariate model was built, again stratified based on the three sets of variables. The interaction between univariate and multivariate results was assessed with the use of the Cochran–Mantel–Haenszel test. The multivariate model was then tested for multicollinearity.

Propensity score analysis was performed to balance possible confounding between the two study groups with regard to the selected variables in order to prevent any bias related to the initial selection of patients for CABG surgery. Propensity scores were computed using a multiple logistic regression model, in which the dependent variable was concomitant ablation and the independent variables were the ones for which the given variable returned an estimated effect of >0.1 change in the respective HR after multiple logistic regression. Regression adjustment was then fitted, resulting in increased precision for the continuous outcome as described by Steyerberg [9]. A greedy match using nearest-neighbor method was used and 1:1 ratio, without replacement, within a specific caliper width of 0.2 SD of the LOGIT of the estimated propensity score. Propensity scores, along with Wald (*χ*^2^), are reported with the corresponding 95% CIs. To verify the balance between concomitant ablation vs. no concomitant ablation groups after propensity score (PS)-matching, the standardized mean differences (SMDs) were computed. For the selected PS-matched population, the univariate and multivariate Cox proportional hazard models were tested again, and statistical differences reported. Overall, late mortality was assessed with the Kaplan–Meier curves fitted before (unadjusted model) and after PS-matching. As a sensitivity analysis to assess the long-term survival following concomitant ablation, patients were stratified according to defined subgroups and the respective models unadjusted and PS-matched redone. The STATA MP v13.0 software (StataCorp, College Station, TX, USA) was used for computations.

## 3. Results

**Figure 1:**
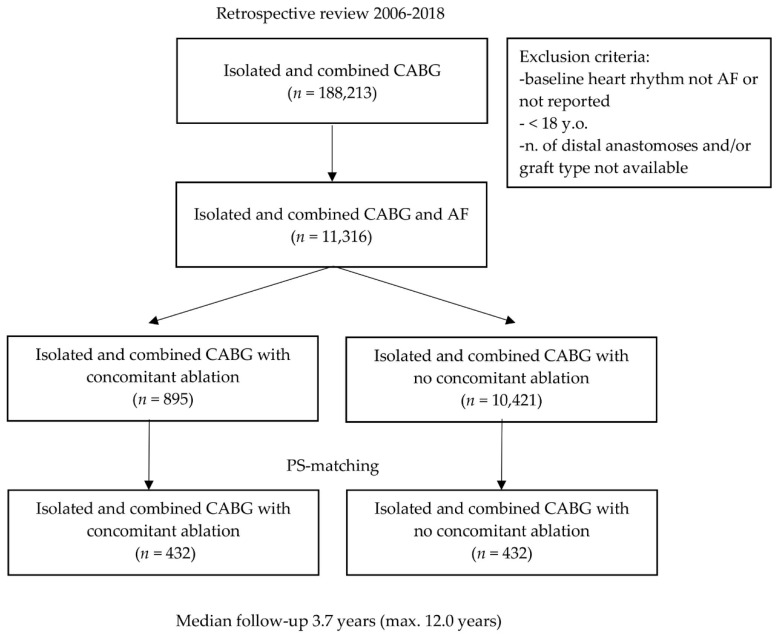
Flow diagram of the current study cohort undergoing isolated and combined CABG with or without concomitant surgical ablation for atrial fibrillation. AF: atrial fibrillation; CABG: coronary artery bypass grafting; y.o.: years old; *n*: number; PS: propensity score.

During the 12-year study period, 193,488 patients undergoing CABG surgery were identified. Among them, 11,316 initially presented with AF. Subjects were divided into concomitant ablation (895 (7.91%) and no concomitant ablation (10,421 (92.09%)), Figure 1).

**Figure 2:**
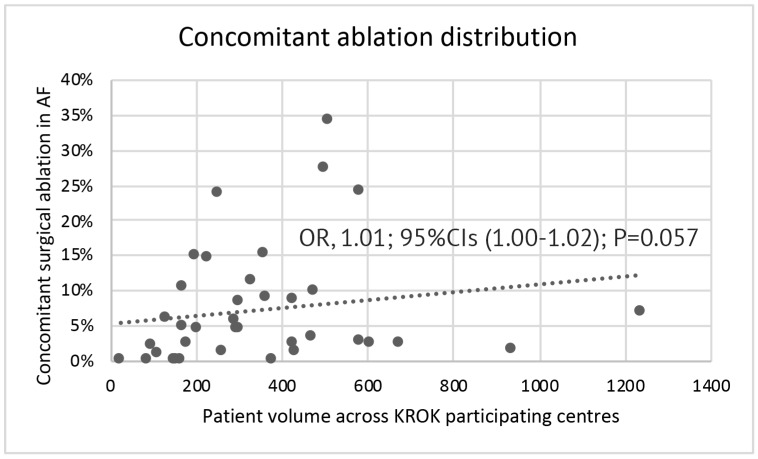
Distribution of concomitant ablation rates across KROK (Polish National Registry of Cardiac Surgery Procedures)-participating centers according to the center’s AF volume. OR: odds ratio; 95% CIs: 95% Confidence Intervals; AF: atrial fibrillation.

Distribution of ablation rates varied across KROK participating centers from 0.0% to 34.2%, and this percentage was borderline statistically associated with the center’s AF volume (odds ratio 1.01; 95% CIs (1.00–1.02); *p* = 0.057, Figure 2).

The median follow-up was four years (3.7 IQR 1.3–6.8). The baseline characteristics, along with the clinical and surgical data of the entire study group before adjustments, are listed in Table S1. Patients in the concomitant ablation group were significantly younger (67.2 ± 7.3 vs. 69.8 ± 7.9 *p* < 0.001) but were at similar baseline surgical risk (mean EuroSCORE 3.22 vs. 3.08; *p* = 0.433). Concomitant ablation patients less frequently presented with insulin-dependent diabetes, (*p* = 0.007), peripheral artery disease (*p* = 0.006), and three-vessel disease (*p* < 0.001).

Regarding the clinical characteristics at the time of the procedure, concomitant ablation subjects less commonly received iv. inotropes (1.1% vs. 2.7%; *p* = 0.005) or had IABP inserted (0.3% vs. 1.7%; *p* = 0.005) as compared to the no concomitant ablation subgroup. The majority of included patients were of elective status (7.322 (64.7%)), of those, 77.1% vs. 63.6% were in the concomitant ablation group. The details on the operative data are further available in Table S2.

### 3.1. Operative and Long-Term Data

Significantly more patients underwent isolated CABG (7.250 (64.1%)) than combined surgeries (35.9%); CPB-CABG was the approach preferred to OPCAB (4.193 (37.1%) vs. 3.057 (27.0%)), with similarly balanced distribution across concomitant ablation vs. no concomitant ablation subsets. Among combined procedures, mitral valve surgery was most frequent (23.6%) followed by the aortic −(14.9%) and tricuspid valve (10.0%). Left internal mammary artery (LIMA) grafts were used in 72.2% and were used more frequently in no concomitant ablation (72.5% vs. 67.8%, *p* = 0.005); pedicled IMA was harvested twice as often as skeletonized IMA (48.0 vs. 24.6%). Complete revascularization was possible in 74.0% of patients and was 7.3% higher in the group undergoing concomitant ablation (*p* < 0.001), while arterial anastomoses accounted for 41.0% of all distal anastomoses, and the total arterial revascularization was achieved in 17.6%.

The median (IQR) HLoS was 10 (8–15) days and the ICU stay was 2.05 (1.13–3.62) days. The HLoS was longer in the concomitant ablation (standardized mean differences (95% CIs) −0.121 (−0.190, −0.053), *p* < 0.001) and so was the length of ICU stay (standardized mean differences (95% CIs) −0.211 (−0.279, −0.143), *p* < 0.001).

**Figure 3:**
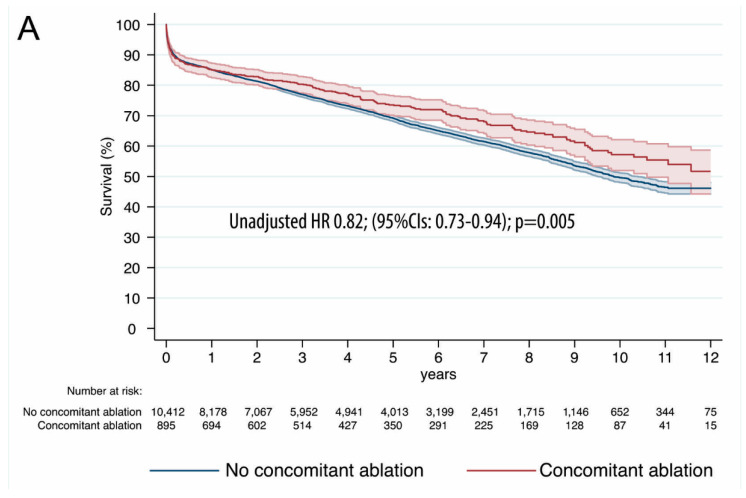

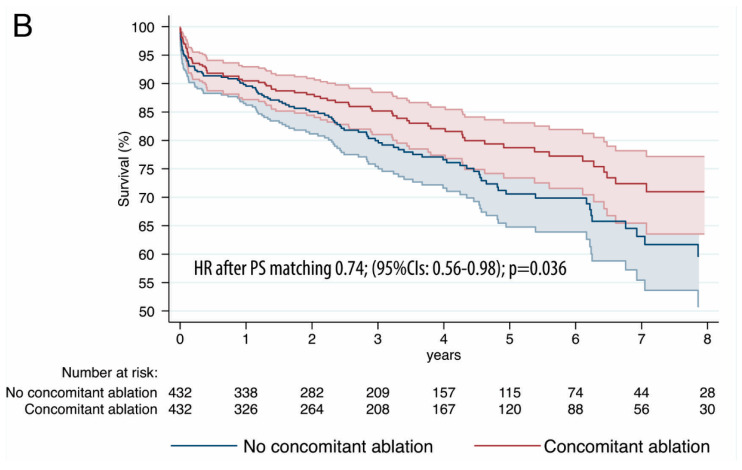
Unadjusted (**A**) and PS-matched (**B**) Kaplan–Meier survival curves between the two groups: concomitant ablation vs. no concomitant ablation. Hazard Ratios and respective 95% Confidence Intervals in the concomitant ablation as compared to no concomitant ablation.

Within investigating the follow-up, the unadjusted HR for the long-term survival favored CABG with concomitant ablation: 0.82; 95% CIs (0.73–0.94); *p* = 0.005 (Figure 3A).

### 3.2. Propensity Score Analysis

One-to-one propensity score-matched analysis resulted in 432 pairs with similar baseline characteristics and operative covariates (Table 1 and Table 2). A list of variables contributing to PS along with the respective propensity scores is available as Table S3. A detailed analysis of standardized mean differences (SMDs) before and after propensity score matching, comparing the covariate values for patients undergoing concomitant ablation vs. no concomitant ablation (Figure S1) revealed that the SMDs for the measured covariates were mostly < 0.1, suggesting covariate balance across groups. PS-matched late survival was estimated for HR: 0.74; (95% CIs: 0.56–0.98); *p* = 0.036 (Figure 3B). The rates of in-hospital complications before- and adjusted for PS were similar between the two groups and are reported in Table S4 and Table 3, respectively.

### 3.3. Sensitivity and Subgroup Analyses

**Figure 4:**
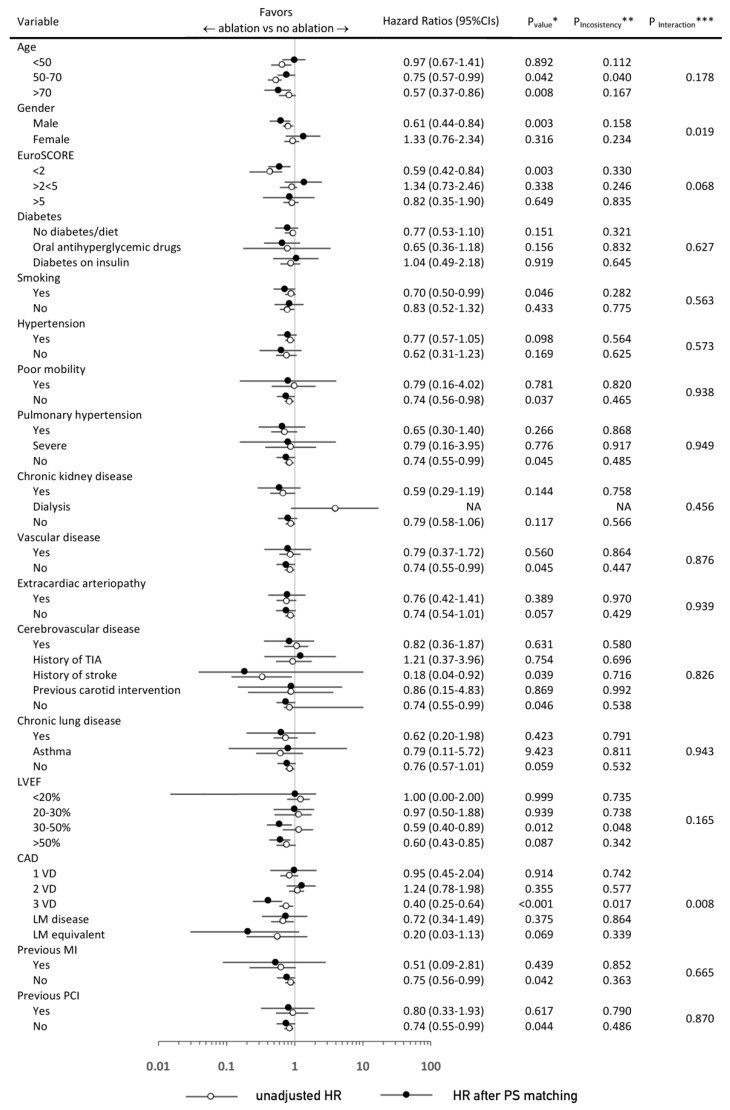
* *p*-value for the treatment effect ** *p*-value for the interaction between pre- and post-PS (Propensity score)-matching estimates *** *p*-value for the interaction between subgroup components (after PS-matching). Hazard Ratios and 95% Confidence Intervals for death from any cause in the concomitant ablation vs. no concomitant ablation groups according to selected preoperative baseline characteristics. CABG: coronary artery bypass grafting; TIA: transient ischemic attack; LVEF: left ventricle ejection fraction; CAD: coronary artery disease; VD: vessel disease; LM: left main; MI: myocardial infarction; PCI: percutaneous coronary intervention; PS: propensity score; NA: not available.

**Figure 5:**
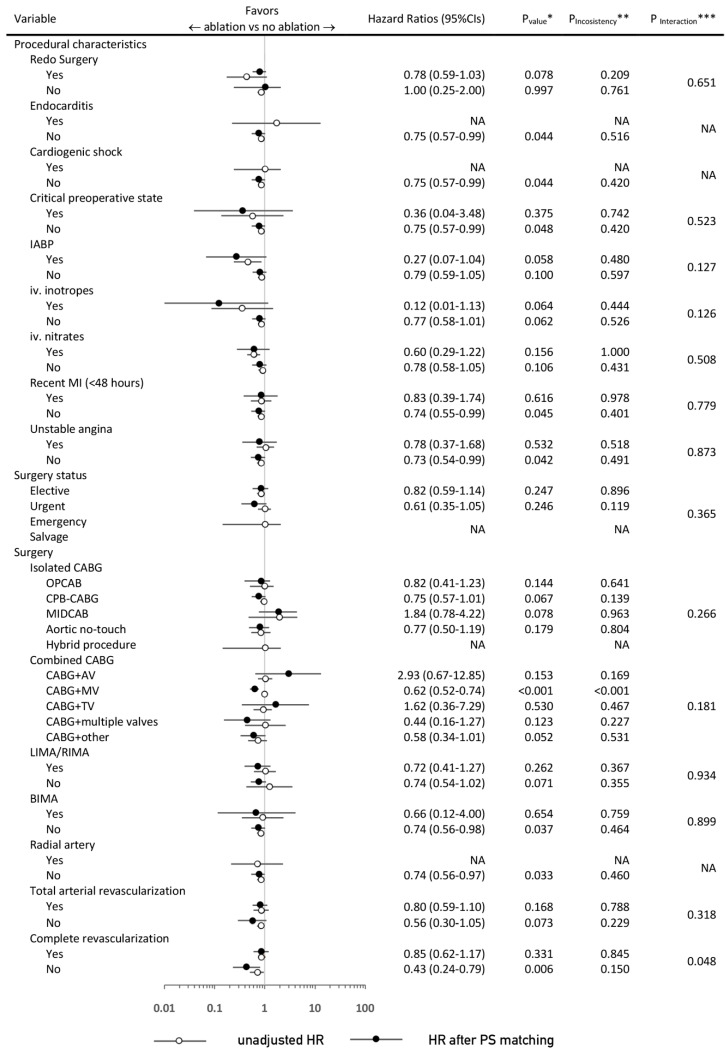
Hazard Ratios and 95% Confidence Intervals for death from any cause in the concomitant ablation vs. no concomitant ablation according to selected procedural characteristics. IABP: intra-aortic balloon pump; iv: intravenous; OPCAB: off-pump coronary artery bypass; CPB: cardiopulmonary bypass; MIDCAB: minimally invasive direct coronary artery bypass; AV: aortic valve; MV: mitral valve; TV: tricuspid valve; LIMA/RIMA/BIMA: left/right/bilateral internal mammary artery. The remaining abbreviations are the same as those in the Figure 4 caption.

The number of subgroup analyses were performed for comparison of concomitant ablation vs. no concomitant ablation before and after PS-matching (Figure 4 and Figure 5) with respect to the primary endpoint, late survival. In these analyses, several significant interactions with baseline (Figure 4) or procedural (Figure 5) variables were demonstrated—before PS-matching, there was a significantly greater extent of benefit in the concomitant ablation arm in a subgroup of patients of male gender (*p* = 0.004) younger than 50 Years old (y.o.) (*p* = 0.013) with EuroSCORE < 2 (*p* < 0.001), without pulmonary hypertension (*p* = 0.009), and in those without previous MI (*p* = 0.047) and PCI (*p* = 0.004).

After PS-matching, the direction of benefit with concomitant ablation was maintained across subgroups of patients, yet was particularly present in patients with lower baseline surgical risk such as those with EuroSCORE < 2, preserved ejection fraction, and without comorbidities. Operatively, the greatest survival benefit in the concomitant ablation group was appraised in CABG with MV surgery (HR: 0.62; (95% CIs: 0.52–0.74); *p* < 0.001), and the absence of arterial revascularization (*p* = 0.071 for the absence of LIMA/RIMA graft; *p* = 0.037 for the absence of BIMA grafts; *p* = 0.033 for the absence of radial artery graft; and *p* = 0.073 for the absence of TAR).

In cases of incomplete revascularization, surgical ablation concomitant to CABG was associated with 57% improved survival as compared to CABG without ablation (HR: 0.43; (95% CIs: 0.24–0.79); *p* = 0.006). The detailed analysis, with reporting for both the univariate and multivariate Cox proportional hazards models, is appended as Table S5.

## 4. Discussion

The presence of preoperative AF in patients undergoing CABG, albeit much less than in patients admitted for isolated MV surgery, reaches up to 6% and accounts for substantial morbidity shortly after the procedure and in the long-term [1,3,5]. While it would, therefore, be advisable to consider performing a concomitant surgical ablation procedure if patients in AF require surgical revascularization, and indeed, class IB recommendation is assigned for surgical ablation at the time of isolated and combined CABG to restore SR in patients with AF [1], partly because of an inherent lack of short-term safety outcome and long-term survival data, and partly because the Cox-Maze III and IV are technically difficult and demanding, this approach is seldom being undertaken [6,10].

In the most recent report from the STS database [2], in patients with documented AF (*n* = 86,941), the overall prevalence of surgical ablation was 48.3%; in this cohort, the most common operation performed was isolated CABG (33.1%), but that had the lowest rate of concomitant ablation (33.0%). Mitral valve surgery with or without CABG was performed in 25.0% of patients but had the highest rate of ablation (68.4%). In the current analysis of the KROK registry, of the 11,316 patients initially presenting with AF, only 895 (7.91%) underwent concomitant surgical ablation, while 10,421 (92.09%) did not, with the percentage dependent on the participating center’s AF volume. Partially reflecting on current overseas trends of that of AF patients undergoing MV surgery, they are at least twice as likely to be granted surgical ablation as compared to their CABG counterparts, another analysis of the KROK registry this time focusing on mitral valve and concomitant surgical ablation demonstrated the prevalence of the latter estimated at 21.5% [7]. Unlike the CABG setting, robust and conclusive data to support the strategy of surgical ablation in MV surgery already exist [11,12]. Indeed, among sparse randomized evidence for the safety and efficacy of surgical ablation for AF at the time of CABG is the PRAGUE 12 trial [13], which randomized 224 patients with AF scheduled for valve and coronary surgery to planned surgery + ablation or planned surgery alone (51.8% isolated or combined CABG), with the primary efficacy outcome being the SR presence during a 24 h electrocardiogram after one year. Interestingly, ECGs revealed SR in 60.2% of ablated patients vs. 35.5% in the group undergoing surgery alone (*p* = 0.002). The combined safety endpoint of death/MI/stroke/AKI (Acute kidney injury) at 30 days occurred in 10.3% vs. 14.7% in surgery + ablation vs. surgery alone, but the study was obviously underpowered for hard clinical endpoints. Additionally, Pokushalov et al. [14] randomized 35 patients to undergo CABG alone or CABG with concomitant epicardial pulmonary vein isolation (PVI). At the 18-month follow-up after surgery, 16 (89%) patients in the CABG + PVI group were AF-free (i.e., AF% < 0.5%) vs. 8 (47%) in the CABG only group (log-rank test, *p* = 0.007). Similar low relapse rates were found by Cherniavsky et al. [15] in a report that randomized 95 patients with persistent AF and CAD to undergo open-heart surgery combined with intraoperative irrigated radiofrequency ablation— CABG + PVI, *n* = 31; CABG + MiniMAZE, *n* = 30; and isolated CABG, *n* = 34. At 14 months, loop recorder-determined freedom from AF was 80% in the CABG + PVI group, 86.2% in the CABG + MM group, and 44.1% in the CABG alone group.

Since there exists some evidence regarding remote effectiveness of surgical ablation concomitant to CABG surgery, the focus of the current analysis was, instead, to address the late survival in AF patients undergoing coronary surgery with or without surgical ablation. Within 12 years of study duration, we found the unadjusted hazard ratio for long-term survival favoring CABG with surgical ablation—HR 0.82; 95% CI (0.73–0.94); *p* = 0.005—with the benefit numerically more prominent after rigorous matching for PS (HR: 0.74; (95% CIs: 0.56–0.98); *p* = 0.036). To our knowledge, the current report is the first to address long-term survival after isolated and combined CABG with or without concomitant ablation on such scale. Importantly, the long-term survival benefit was not compromised in the early follow-up; there were no differences in neither early and 30-day mortality, nor in-hospital complications after PS matching. These results perfectly reflect recent analysis by Rankin et al. [16] whose important findings were that for patients with AF who underwent CABG, no significant difference in risk-adjusted rates of perioperative death and complications were observed with or without surgical ablation (5.3% vs. 5.2%; adjusted OR 1.15, *p* = 0.19). Conversely to the above Medicare analysis that found survival curves diverging in favor of surgical ablation at 90 days postop, in the current analysis, survival benefit becomes apparent slightly before two years follow-up, in turn, suggesting other than surgery-related causes of death in the non-ablated patients undergoing CABG. More importantly, the achieved survival benefit was maintained across a number of sensitivity subgroup analyses dividing patients into opposing category strata; the only significant interactions suggestive of the different magnitudes of treatment effects were found in the categories: sex, EuroSCORE, the extent of CAD, and completeness of revascularization, with more pronounced effects in men, low risk (EuroSCORE < 2), three-vessel disease, and incomplete revascularization patients. The current analysis also puts into a wider perspective the findings of another recent report from the KROK registry [17]; however, focusing solely on patients undergoing surgical ablation concomitant to isolated CABG. In the long term, surgical ablation was associated with a significant 33% improved overall survival rate with the benefit of ablation sustained in a subgroup analysis but most pronounced in lower risk older patients, with three-vessel disease, history of a cerebrovascular accident, and preserved left ventricular function, and those undergoing on-pump CABG. The picture is now complemented by the fact that surgical ablation is even more beneficial when the MV procedure is performed as well. In fact, independent from baseline characteristics, surgical ablation was associated with nearly 40% improved late survival in a subgroup of patients undergoing concomitant MV surgery (HR: 0.62; (95% CIs: 0.52–0.74); *p* < 0.001). In line with these findings is another report from the Northern New England Cardiovascular Disease Study Group Cardiac Surgery Registry [18] that enrolled 20,407 CABG, valve, or CABG + valve patients undergoing surgical ablation or not. With the prevalence estimated at 23.1%, within a 2.6-year follow-up, surgical ablation was associated with 31% improved survival in the CABG + valve subgroup (HR = 0.69; (95%CI = 0.51–0.92), *p* = 0.013) in an adjusted analysis.

An unexpected, yet possibly the most important finding of the current analysis is the nearly 60% significantly improved survival in patients undergoing surgical ablation in whom complete revascularization was not achieved. Incomplete revascularization (IR) remains among the greatest threats of CABG surgery, and is associated with significantly impaired early and late survival; in the National Institute for Cardiovascular Outcomes Research registry [19] with 13,701 patients analyzed, the IR increased risk of death is over two-fold regardless of its extent (HR 2.15; 95% CI 1.57–2.93). Why surgical ablation is protective against IR’s complications and translates into such improved survival remains to be answered. We can speculate that in the critically ill cardiac surgery patient, the loss of SR and atrial contribution to cardiac output can lead to significant hemodynamic instability [20]. In the scenario of IR and increased oxygen myocardial demand, adequate flow through the remaining grafts will be of paramount importance. Another potential explanation lies in an increase in the dosing of antiplatelet drugs in the case of IR. In fact, it might be reasonable that secondary pharmacological treatment could compensate surgical IR in diffuse coronary artery disease of the non-LAD territories since more aggressive pharmacological approach with statins and full antiplatelet therapy has ameliorated the outcome of candidates to IR. In the remote follow-up, however, “CABG-IR” patients are subject to consecutive deferred PCI for non-revascularized territory. By prolonging DAPT (Dual antiplatelet therapy) together with anticoagulant treatments for AF, these patients are put at risk for bleeding events, which may have contributed to worse survival in the CABG alone group, as seen in our analysis.

### Limitations

Limitations of the KROK registry have been described before [7,17,21]. First and foremost, it is an observational study, and the lack of randomization means that patients in the concomitant ablation group were undoubtedly healthier than their no concomitant ablation counterparts. We attempted to minimize this selection bias by propensity matching patients, and we found that after matching, the patients in the two groups had very similar profiles with respect to the numerous risk factors available in the registry and far beyond EuroSCORE’s single components. The KROK registry, which has now collected the data of over 600,000 procedures, was designed to address in detail only in-hospital data on patients and outcomes; the long-term effectiveness of concomitant ablation and, therefore, conversions to SR and maintenance rates are not available from the registry itself. More importantly, the registry does not record the rates of long-term strokes and other complications. Indeed, an upgrade of the registry allowing for the transfer of the data from the NHS records to the KROK registry other than survival is currently underway. The exact causes of death (cardiovascular vs. non-cardiovascular) were available only in < 5% of the NHS records and precluded detailed analyses. We could not account for left atrial appendage (LAA) closure rates and ablation- durations, techniques, and immediate success rates since these were not obligatory to complete during the registry conception, and these data are incomplete. The finding on significantly improved survival in patients undergoing concomitant surgical ablation in whom complete revascularization was not achieved should be interpreted with caution. Indeed, only around 80 patients in each group did not undergo complete revascularization after matching for propensity scores, and these are particularly sensitive to bias and lack of statistical power. Completeness of the revascularization index (CRI) was further calculated based on the difference between the number of coronary grafts and the number of diseased coronary artery systems, as reported in the KROK database. While CRI reached an adjusted 82% in the current analysis, these rates may be underscored since they do not represent intended vs. performed anastomoses ratio. Finally, the registry does not collect the long-term medication regimens; while all participating centers ensured that patients were treated according to structured protocols adhering to the ECS/EACTS (European Society of Cardiology/European Association for Cardio-Thoracic Surgery) guidelines for post-operative cardiac surgery care effective at the time of surgery, we could not account for patients’ long-term adherence (i.e., beta-blocker, statin, antiplatelet agents, anticoagulants (warfarin or non-vitamin K dependent anticoagulants), etc.) and in particular, between the two groups. The possible differences in this regard could further influence the remote outcomes.

## 5. Conclusions

Concomitant surgical ablation for atrial fibrillation in patients undergoing isolated and combined coronary artery bypass grafting is safe and feasible; in unadjusted analysis and after rigorous propensity matching, surgical ablation was associated with improved long-term survival as well, and in particular, in concomitant mitral valve surgery and in cases when complete coronary revascularization was not achieved.

## Figures and Tables

**Table 1:** Preoperative characteristics after PS-matching.

Variable	PS-Matched Patients			
	Total (864)	Concomitant Ablation (432)	No Concomitant Ablation (432)	*p*-Value
Age years (median (IQR))	68.6 (63.19–73.36)	68.6 (63.2–73.6)	68.5 (63.1–73.11)	0.714
<50	10 (1.2%)	5 (1.2%)	5 (1.2%)	1.000
50–70	487 (56.4%)	246 (56.9%)	241 (55.8%)	0.732
>70	367 (42.5%)	181 (41.9%)	186 (43.1%)	0.731
Gender				
Male	657 (76.0%)	328 (75.9%)	329 (76.2%)	0.937
Female	207 (24.0%)	104 (24.1%)	103 (23.8%)	
EuroSCORE (median (IQR))	1.32 (0.89–2.72)	1.38 (0.90–2.97)	1.28 (0.88–2.48)	0.092
<2	568 (65.7%)	272 (63.0%)	296 (68.5%)	0.086
2–5	204 (23.6%)	110 (25.5%)	94 (21.8%)	0.201
>5	92 (10.6%)	50 (11.6%)	42 (9.7%)	0.378
Diabetes	330 (38.2%)	154 (35.6%)	176 (40.7%)	0.124
Insulin dependent	105 (12.2%)	46 (10.6%)	59 (13.7%)	0.177
Smoking	538 (62.3%)	261 (60.4%)	277 (64.1%)	0.261
Hypertension	752 (87.0%)	377 (87.3%)	375 (86.8%)	0.839
Hyperlipidemia	535 (61.9%)	259 (60.0%)	276 (63.9%)	0.234
Poor mobility	46 (5.3%)	20 (4.6%)	26 (6.0%)	0.365
BMI (median (IQR))	28.25 (25.46–31.23)	28.08 (23.32–30.92)	28.38 (25.57–31.57)	0.919
Pulmonary hypertension	113 (13.1%)	50 (11.6%)	63 (14.6%)	0.190
Severe (PA systolic > 55 mmHg)	19 (2.2%)	10 (2.3%)	9 (2.1%)	0.817
Renal impairment	309 (35.8%)	152 (35.2%)	157 (36.3%)	0.723
moderate (CC > 50 & <85)	229 (26.5%)	117 (27.1%)	112 (25.9%)	0.700
severe (CC < 50)	80 (9.3%)	35 (8.1%)	45 (10.4%)	0.241
dialysis (regardless of CC)	4 (0.5%)	2 (0.5%)	2 (0.5%)	1.000
Peripheral artery disease	119 (13.8%)	57 (13.2%)	62 (14.4%)	0.622
Cerebrovascular disease	81 (9.4%)	35 (8.1%)	46 (10.6%)	0.201
Stroke	30 (3.5%)	16 (3.7%)	14 (3.2%)	0.710
TIA	207 (24.0%)	104 (24.1%)	103 (23.8%)	0.936
Carotid intervention	9 (1.0%)	3 (0.7%)	6 (1.4%)	0.325
Chronic lung disease	63 (7.3%)	32 (7.4%)	31 (7.2%)	0.896
Asthma	26 (3.0%)	12 (2.8%)	14 (3.2%)	0.691
LVEF (%) (median (IQR)) *	50.0 (40.0–55.0)	50.0 (40.0–57.0)	49.0 (40.0–55.0)	0.642
<20%	31 (3.6%)	13 (3.1%)	18 (4.2%)	0.129
21–30%	107 (12.4%)	60 (13.9%)	47 (10.9%)	0.180
31–50%	421 (48.7%)	198 (45.8%)	223 (51.6%)	0.088
>50%	305 (35.3%)	161 (37.2%)	144 (33.3%)	0.226
CAD				
1 VD	200 (23.1%)	97 (22.5%)	103 (23.8%)	0.062
2 VD	357 (41.3%)	181 (41.9%)	176 (40.7%)	0.713
3 VD	307 (35.5%)	154 (35.6%)	153 (35.4%)	0.943
LM disease	115 (13.3%)	58 (13.4%)	57 (13.2%)	0.902
Previous MI	30 (3.5%)	16 (3.7%)	14 (3.2%)	0.710
Previous PCI	100 (11.6%)	54 (12.5%)	46 (10.6%)	0.396

* missing data; CABG: coronary artery bypass grafting; PS: propensity score; IQR: interquartile range; BMI: body mass index; PA: pulmonary artery; CC: creatinine clearance; TIA: transient ischemic attack; LVEF: left ventricle ejection fraction; CAD: coronary artery disease; VD: vessel disease; MI: myocardial infarction; PCI: percutaneous coronary intervention.

**Table 2:** Operative characteristics after PS-matching.

Variable	PS-Matched Patients			
	Total (864)	Concomitant Ablation (432)	No Concomitant Ablation (432)	*p*-Value
***Procedural Characteristics***				
Redo surgery	18 (2.1%)	9 (2.1%)	9 (2.1%)	1.000
Endocarditis	2 (0.2%)	1 (0.2%)	1 (0.2%)	1.000
Cardiogenic chock	2 (0.2%)	1 (0.2%)	1 (0.2%)	1.000
Critical preoperative state	10 (1.2%)	4 (0.9%)	6 (1.4%)	0.528
IABP	5 (0.6%)	2 (0.5%)	3 (0.7%)	0.656
iv. inotropes	10 (1.2%)	5 (1.2%)	5 (1.2%)	1.000
iv. nitrates	116 (13.4%)	57 (13.2%)	59 (13.7%)	0.842
***Urgency***				
Elective	633 (73.3%)	316 (73.1%)	317 (73.4%)	0.939
Urgent	216 (25.0%)	109 (25.2%)	107 (24.8%)	0.875
Emergency	12 (1.4%)	7 (1.6%)	5 (1.2%)	0.563
Salvage	0 (0.0%)	0 (0.0%)	0 (0.0%)	1.000
***Surgery***				
Isolated CABG	644 (74.5%)	322 (75.5%)	322 (74.5%)	1.000
OPCAB *	347 (40.1%)	170 (39.4%)	177 (41.0%)	0.627
CPB-CABG *	297 (34.4%)	152 (35.2%)	145 (33.6%)	0.616
MIDCAB	26 (3.0%)	13 (3.0%)	13 (3.0%)	1.000
Aortic no-touch	92 (10.6%)	44 (10.2%)	48 (11.1%)	0.659
Hybrid procedure	1 (0.1%)	1 (0.2%)	0 (0.0%)	0.501
Combined CABG	220 (25.5%)	110 (25.5%)	110 (25.5%)	1.000
CABG + MV	74 (8.6%)	35 (8.1%)	39 (9.0%)	0.496
CABG + AV	55 (6.4%)	29 (6.7%)	26 (6.0%)	0.676
CABG + TV	38 (4.4%)	17 (3.9%)	21 (4.9%)	0.508
CABG + multiple valves	23 (2.7%)	10 (2.3%)	13 (3.0%)	0.527
CABG + other	30 (3.5%)	19 (4.4%)	11 (2.5%)	0.141
***Grafts and Anastomoses***				
LIMA	638 (73.8%)	317 (73.4%)	321 (74.3%)	0.757
RIMA	22 (2.5%)	9 (2.1%)	13 (3.0%)	0.391
BIMA	19 (2.2%)	8 (1.9%)	11 (2.5%)	0.488
Pedicled IMA *	438 (50.7%)	218 (50.5%)	220 (50.9%)	0.890
Skeletonized IMA *	221 (25.6%)	112 (25.9%)	109 (25.2%)	0.815
Radial artery	22 (2.5%)	8 (1.9%)	14 (3.2%)	0.201
Arterial anastomoses	282 (40.0%)	145 (40.8%)	137 (39.1%)	0.645
Venous anastomoses	423 (60.0%)	210 (59.2%)	213 (60.9%)	0.645
Sequential anastomoses	69 (9.8%)	38 (10.7%)	31 (8.9%)	0.409
Composite anastomoses	61 (7.1%)	27 (6.3%)	34 (7.9%)	0.353
Total arterial revascularization	181 (20.9%)	80 (18.5%)	101 (23.4%)	0.080
*Completeness of revascularization*	708 (81.9%)	352 (81.5%)	356 (82.4%)	0.724

* missing data; CABG: coronary artery bypass grafting; PS: propensity score; IABP: intra-aortic balloon pump; iv: intravenous; OPCAB: Off-Pump Coronary Artery Bypass; CPB: cardiopulmonary bypass; MIDCAB: Minimally Invasive Direct Coronary Artery Bypass; MV: mitral valve; AV: aortic valve; TV: tricuspid valve; LIMA/RIMA/BIMA: Left/Right/Bilateral Internal Mammary Artery.

**Table 3:** In-hospital complications after PS-matching.

	PS-Matched Patients			
	Concomitant Ablation (432)	No Concomitant Ablation (432)	Risk Ratio (95% CIs)	*p*-Value
Early postoperative mortality	4 (0.9%)	0 (0.0%)	9.00 (0.49–166.65)	0.140
30-day mortality	21 (4.9%)	31 (7.2%)	0.67 (0.40–1.16)	0.156
Cardiac tamponade and/or rethoracotomy	35 (7.5%)	30 (8.1%)	1.17 (0.73–1.87)	0.519
Periprocedural MI	5 (1.2%)	3 (0.7%)	1.67 (0.40–6.93)	0.482
Respiratory failure	35 (8.1%)	20 (4.6%)	1.75 (1.03–2.98)	0.040
Prolonged ICU stay	5 (1.2%)	8 (1.9%)	0.63 (0.21–1.90)	0.406
Neurologic complications	12 (2.8%)	9 (2.1%)	1.33 (0.57–3.13)	0.509
Pulmonary embolism	3 (0.7%)	0 (0.0%)	7.00 (0.36–135.11)	0.198
Multiogran failure	10 (2.3%)	7 (1.6%)	1.43 (0.55–3.72)	0.465
Gastrointestinal complications	5 (1.2%)	6 (1.4%)	0.83 (0.26–2.71)	0.762
Acute kidney failure	1 (0.2%)	0 (0.0%)	3.00 (0.12–73.44)	0.501
Sternal wound infection	13 (3.0%)	15 (3.5%)	0.87 (0.42–1.80)	0.701
ECMO	1 (0.2%)	0 (0.0%)	3.00 (0.12–73.44)	0.501
VAD	1 (0.2%)	0 (0.0%)	3.00 (0.12–73.44)	0.501

CABG: coronary artery bypass grafting; PS: propensity score; CIs: confidence intervals; MI: myocardial infarction; ICU: intensive care unit; ECMO: extracorporeal membrane oxygenation; VAD: ventricle assist device.

## Supplementary Materials

The following are available online at https://www.mdpi.com/2077-0383/9/5/1345/s1, Figure S1: Standardized differences before and after propensity score matching comparing covariate values for patients undergoing concomitant surgical ablation vs. no concomitant surgical ablation, Table S1: Preoperative characteristics before PS-matching, Table S2: Operative characteristics before PS-matching, Table S3: Propensity scores, Table S4: In-hospital outcomes before PS-matching, Table S5: Cox proportional hazard univariate and multivariate model estimates before and after PS-matching.
