# Supplementary material for: Long-Term Survival Following Surgical Ablation for Atrial Fibrillation Concomitant to Isolated and Combined Coronary Artery Bypass Surgery—Analysis from the Polish National Registry of Cardiac Surgery Procedures (KROK)
Source: J Clin Med. 2020 May 4;9(5):1345. doi: 10.3390/jcm9051345 (PMC7290935; doi:10.3390/jcm9051345)
Supplement: Supplementary file 1 [file jcm-09-01345-s001.pdf]

Table S1. Preoperative characteristics before PS-matching.

Variable	All patients			
	**Total (11,316)**	**Concomitant Ablation (895)**	**No Concomitant Ablation (10,421)**	***p*-Value**
	Baseline Characteristics			
Age years (median (IQR))	70.3 (64.1–75.6)	67.5 (62.2–72.5)	70.5 (64.2–75.8)	<0.001
< 50	103 (0.9%)	10 (1.1%)	93 (0.9%)	0.497
50–70	5,415 (47.9%)	557 (62.2%)	4,858 (46.6%)	<0.001
> 70	5,798 (51.2%)	328 (36.6%)	5,470 (52.5%)	<0.001
Gender				
Male	8197 (72.4%)	650 (72.6%)	7545 (72.4%)	0.895
Female	3118 (27.6%)	245 (27.4%)	2873 (27.6%)	
EuroSCORE (median (IQR))	1.53 (0.95–3.12)	1.55 (0.95–3.18)	1.52 (0.95–3.11)	0.433
< 2	6913 (61.1%)	535 (59.8%)	6365 (61.1%)	0.450
2–5	2792 (24.7%)	232 (25.9%)	2560 (24.6%)	0.363
> 5	1624 (14.4%)	128 (14.3%)	1496 (14.4%)	0.965
Diabetes	4317 (38.1%)	315 (35.2%)	4002 (38.4%)	0.064
Insulin dependent	1780 (15.7%)	112 (12.5%)	1668 (16.0%)	0.007
Smoking	6674 (59.0%)	568 (63.5%)	6106 (58.6%)	0.003
Hypertension	9968 (88.1%)	759 (84.8%)	9209 (88.4%)	0.005
Hyperlipidemia	6909 (61.1%)	521 (58.2%)	6388 (61.3%)	0.079
Poor mobility	513 (4.5%)	33 (3.7%)	480 (4.6%)	0.208
BMI (median [IQR])	28.0 (25.10–31.09)	28.31 (25.22–30.93)	27.94 (25.09–3.11)	0.163
Pulmonary hypertension	906 (8.0%)	114 (12.7%)	792 (7.6%)	<0.001
Severe (PA systolic > 55 mmHg)	175 (1.5%)	16 (1.8%)	159 (1.5%)	0.542
Renal impairment	3350 (29.6%)	247 (27.6%)	3103 (29.8%)	0.012
moderate (CC > 50 & < 85)	2586 (22.9%)	193 (21.6%)	2393 (23.0%)	0.343
severe (CC < 50)	764 (6.8%)	54 (6.0%)	710 (6.8%)	0.374
dialysis (regardless of CC)	58 (0.5%)	3 (0.3%)	55 (0.5%)	0.443
Peripheral artery disease	1692 (15.0%)	105 (11.7%)	1.587 (15.2%)	0.006
Cerebrovascular disease	1046 (9.2%)	71 (7.9%)	975 (9.4%)	0.162

Stroke	416 (3.7%)	28 (3.1%)	388 (3.7%)	0.366
TIA	3118 (27.6%)	245 (27.4%)	2873 (27.6%)	0.900
Carotid intervention	90 (0.8%)	5 (0.6%)	85 (0.8%)	0.410
Chronic lung disease	1010 (8.9%)	78 (8.7%)	932 (8.9%)	0.818
Asthma	439 (3.9%)	31 (3.5%)	408 (3.9%)	0.503
LVEF (%) (median [IQR]) *	50.0 (40.0-55.0)	49.0 (40.0-55.0)	50.0 (40.0-55.0)	0.276
< 20%	189 (1.9%)	11 (1.4%)	178 (2.0%)	0.260
21–30%	1016 (10.3%)	75 (9.5%)	941 (10.4%)	0.419
31–50%	5043 (51.2%)	407 (51.5%)	4636 (51.2%)	0.893
> 50%	3595 (36.5%)	298 (37.7%)	3297 (36.4%)	0.481
CAD				
1 VD	2472 (21.9%)	257 (28.7%)	2215 (21.3%)	< 0.001
2 VD	3026 (26.7%)	255 (29.5%)	2771 (26.6%)	0.057
3 VD	5818 (51.4%)	383 (42.8%)	5435 (52.2%)	< 0.001
LM disease	2435 (21.5%)	124 (13.8%)	2311 (22.2%)	< 0.001
Previous MI	416 (3.7%)	28 (3.13%)	388 (3.7%)	0.364
Previous PCI	1080 (9.5%)	83 (9.3%)	997 (9.6%)	0.774

* missing data; CABG: coronary artery bypass grafting; PS: propensity score; IQR: interquartile range; BMI: body mass index; PA: pulmonary artery; CC: creatinine clearance; TIA: transient ischemic attack; LVEF: left ventricle ejection fraction; CAD: coronary artery disease; VD: vessel disease; MI: myocardial infarction; PCI: percutaneous coronary intervention.

Table S2. Operative characteristics before PS-matching.

**Variable**	**All patients**			
	**Total (11,316)**	**Concomitant Ablation (895)**	**No Concomitant Ablation (10,421)**	***p*-Value**
Procedural Characteristics				
Redo surgery	293 (2.6%)	23 (2.6%)	270 (2.6%)	0.970
Endocarditis	64 (0.6%)	2 (0.2%)	62 (0.6%)	0.172
Cardiogenic chock	140 (1.2%)	3 (0.3%)	137 (1.3%)	0.019
Critical preoperative state	237 (2.1%)	6 (0.7%)	231 (2.2%)	0.004
IABP	185 (1.6%)	3 (0.3%)	182 (1.7%)	0.005
iv. inotropes	296 (2.6%)	10 (1.1%)	286 (2.7%)	0.005

iv. nitrates	1707 (15.1%)	214 (23.9%)	1493 (14.3%)	< 0.001
Urgency				
Elective	7322 (64.7%)	690 (77.1%)	6632 (63.6%)	< 0.001
Urgent	3648 (32.2%)	195 (21.8%)	3453 (33.1%)	< 0.001
Emergency	216 (1.9%)	7 (0.8%)	209 (2.0%)	0.014
Salvage	23 (0.2%)	0 (0.0%)	23 (0.2%)	0.328
Surgery				
Isolated CABG	7250 (64.1%)	556 (62.1%)	6694 (64.2%)	0.206
OPCAB *	4193 (37.1%)	399 (44.6%)	3794 (36.4%)	< 0.001
CPB-CABG *	3057 (27.0%)	157 (17.5%)	2900 (27.8%)	< 0.001
MIDCAB	124 (1.1%)	13 (1.5%)	111 (1.1%)	0.287
Aortic no-touch	1349 (11.9%)	97 (10.8%)	1252 (12.0%)	0.297
Hybrid procedure	31 (0.3%)	2 (0.2%)	29 (0.3%)	0.764
Combined CABG	4066 (35.9%)	339 (37.9%)	3727 (35.8%)	0.206
CABG+MV	2672 (23.6%)	226 (25.3%)	2446 (23.5%)	0.225
CABG+AV	1689 (14.9%)	139 (15.5%)	1550 (14.9%)	0.596
CABG+TV	1132 (10.0%)	96 (10.7%)	1036 (9.9%)	0.451
CABG+multiple valves	1256 (11.2%)	110 (12.3%)	1156 (11.1%)	0.275
CABG+other	1264 (11.2%)	90 (10.1%)	1174 (11.3%)	0.270
Grafts and Anastomoses				
LIMA	8167 (72.2%)	607 (67.8%)	7560 (72.5%)	0.005
RIMA	266 (2.4%)	19 (2.1%)	247 (2.4%)	0.640
BIMA	230 (2.0%)	16 (1.8%)	214 (2.1%)	0.589
Pedicled IMA*	5430 (48,0%)	416 (46.5%)	5014 (48.1%)	0.348
Skeletonized IMA*	2786 (24.6%)	197 (22.0%)	2589 (24.8%)	0.059
Radial artery	187 (1.7%)	12 (1.3%)	175 (1.7%)	0.448
Arterial anastomoses	10,212 (41.0%)	836 (42.8%)	9376 (40.8%)	0.090
Venous anastomoses	14,709 (59.0%)	1119 (57.2%)	13,590 (59.2%)	0.101
Sequential anastomoses	2194 (8.8%)	296 (15.1%)	1898 (8.3%)	< 0.001
Composite anastomoses	787 (7.0%)	56 (6.3%)	731 (7.0%)	0.394
Total arterial revascularization	1991 (17.6%)	152 (17.0%)	1839 (17.6%)	0.287

Completeness of revascularization	8373 (74.0%)	722 (80.7%)	7651 (73.4%)	< 0.001

* missing data; CABG: coronary artery bypass grafting; PS: propensity score; IABP: intra-aortic balloon pump; iv: intravenous; OPCAB: Off-Pump Coronary Artery Bypass; CPB: cardiopulmonary bypass; MIDCAB: Minimally Invasive Direct Coronary Artery Bypass; MV: mitral valve; AV: aortic valve; TV: tricuspid valve; LIMA/RIMA/BIMA: Left/Right/Bilateral Internal Mammary Artery.

Table S3. Propensity scores.

Variable	Wald/χ^2	Score	OR	95% CI Lower	95% CI Upper	*p*-Value	logHF
acute kidney injury	1.56078	−0.05974	2.66	1.17	6.02	0.019	−3056.76
cerebrovascular disease	1.78462	−0.08694	1.21	1.03	1.42	0.018	−3057.66
chronic kidney disease-dialysis	8.78690	−1.19539	3.69	1.41	9.67	0.008	−3063.65
chronic lung disease	9.48446	−0.02533	1.49	1.27	1.74	< 0.001	−3056.82
oral antihyperglycemic drugs	2.86451	0.02380	1.24	1.09	1.41	0.001	−3056.87
iv. inotropes	3.70960	−0.29284	1.72	1.24	2.38	0.001	−3058.36
emergency	6.68444	−0.45670	1.79	1.27	2.52	0.001	−3060.28
IABP	3.06909	−0.11857	0.80	0.66	0.96	0.019	−3058.17
diabetes on insulin	24.78087	−0.11815	1.65	1.45	1.87	< 0.001	−3059.26
critical perioperative state	3.76159	−0.41900	1.77	1.24	2.53	0.002	−3059.11
surgery on thoracic aorta	4.59533	−0.07335	1.41	1.05	1.91	0.024	−3056.99
previous PCI	11.98462	−0.08507	0.77	0.63	0.93	0.006	−3057.73
peripheral artery disease	6.63136	−0.13753	1.43	1.25	1.63	< 0.001	−3060.05
pulmonary hypertension-severe	9.91389	−0.03110	2.57	1.78	3.73	< 0.001	−3056.76
normal kidney function (cc > 85 mL/min)	32.30000	−0.02460	0.17	0.14	0.20	< 0.001	−3056.90
severe renal impairment (cc < 50)	25.20942	−0.18582	0.21	0.17	0.27	< 0.001	−3059.50
serum creatinine level	33.17604	−0.34975	0.77	0.70	0.85	< 0.001	−3072.75
smoking	7.47618	−0.10072	1.22	1.11	1.35	< 0.001	−3060.57
transfusions	38.23149	−0.25425	1.39	1.21	1.59	< 0.001	−3075.11
cardiogenic shock	1.54834	−0.26476	1.78	1.10	2.89	0.020	−3057.13

IABP, intra-aortic balloon pump; PCI, percutaneous coronary intervention.

Table S4. In-hospital outcomes before PS-matching.

	All patients			
	**Concomitant Ablation (895)**	**No Concomitant Ablation (10,421)**	**Risk Ratio (95% CIs)**	***p*-Value**
Early postoperative mortality	11 (1.2%)	123 (1.2%)	1.04 (0.56–1.92)	0.897
30-day mortality	43 (4.8%)	573 (5.5%)	0.87 (0.65–1.18)	0.382
Cardiac tamponade and/or rethoracotomy	78 (8.7%)	775 (7.4%)	1.17 (0.94–1.46)	0.163
Periprocedural MI	14 (1.6%)	118 (1.1%)	1.38 (0.80–2.39)	0.249
Respiratory failure	85 (9.5%)	841 (8.1%)	1.18 (0.95–1.46)	0.133
Prolonged ICU stay	20 (2.2%)	192 (1.8%)	1.21 (0.77–1.91)	0.406
Neurologic complications	24 (2.7%)	335 (3.2%)	0.83 (0.55–1.26)	0.384
Pulmonary embolism	6 (0.7%)	61 (0.6%)	1.15 (0.50–2.64)	0.750
Multiogran failure	30 (3.4%)	363 (3.5%)	0.96 (0.67–1.39)	0.837
Gastrointestinal complications	18 (2.0%)	193 (1.9%)	1.09 (0.67–1.75)	0.735
Acute kidney failure	3 (0.3%)	37 (0.4%)	0.94 (0.29–3.06)	0.923
Sternal wound infection	32 (3.6%)	317 (3.0%)	1.18 (0.82–1.68)	0.375
ECMO	3 (0.3%)	23 (0.2%)	1.52 (0.46–5.05)	0.495
VAD	2 (0.2%)	7 (0.1%)	3.33 (0.69–15.99)	0.133

CABG: coronary artery bypass grafting; PS: propensity score; CIs: confidence intervals; MI: myocardial infarction; ICU: intensive care unit; ECMO: extracorporeal membrane oxygenation; VAD: ventricle assist device.

Table S5. Cox proportional hazard univariate and multivariate model estimates before and after PS-matching.

**Model**	**Univariate**				**Multivariate**			
**Estimates**	**Unadjusted Hazard Ratios**		**After PS-Matching Hazard Ratios**		**Unadjusted Hazard Ratios**		**After PS-Matching Hazard Ratios**	
**Variables**	**HR (95% CIs)**	***p***	**HR (95% CIs)**	***p***	**HR (95% CIs)**	***p***	**HR (95% CIs)**	***p***
Age	1.01 (1.01–1.01)	< 0.001	1.01 (1.00–1.01)	< 0.001	1.01 (1.00–1.02)	0.165		
Male	1.12 (1.08–1.17)	< 0.001			1.08 (0.90–1.23)	0.346		

Female	0.88 (0.83–0.92)	< 0.001			0.92 (0.77–1.10)	0.379		
Euroscore	1.00 (1.00–1.01)	0.206			0.99 (0.97–1.02)	0.488		
Diabetes								
No diabetes/diet	0.86 (0.80–0.91)	< 0.001			0.78 (0.57–0.96)	0.016		
Oral antihyperglycemic drugs	1.17 (1.10–1.24)	< 0.001	1.15 (1.08–1.22)	<0.001	1.08 (0.89–1.31)	0.454		
Diabetes on insulin	1.04 (0.98–1.11)	0.220			1.14 (0.90–1.45)	0.277		
Smoking	1.05 (1.00–1.10)	0.036	1.05 (1.00–1.10)	0.038	1.21 (1.04–1.42)	0.017		
Hypertension	1.22 (1.14–1.31)	< 0.001	1.13 (1.06–1.22)	0.001	1.38 (1.09–1.75)	0.007		
Hyperlipidemia	1.10 (1.05–1.15)	< 0.001			1.24 (1.06–1.46)	0.007	1.22 (1.04–1.43)	0.016
Poor mobility	3.42 (3.07–3.81)	< 0.001	2.35 (2.10–2.62)	< 0.001	3.39 (2.45–4.69)	<0.001	3.40 (2.45–4.73)	< 0.001
Pulmonary hypertension	3.88 (3.57–4.22)	< 0.001			2.08 (1.65–2.63)	<0.001		
Severe pulmonary hypertension	2.37 (1.95–2.87)	< 0.001			1.27 (0.73–2.20)	0.396		
Chronic kidney disease	1.78 (1.65–1.93)	< 0.001	1.40 (1.29–1.52)	< 0.001	1.47 (1.12–1.92)	0.005		
Dialysis	1.76 (1.21–2.55)	0.003			1.38 (0.44–4.30)	0.577		
Vascular disease	1.18 (1.10–1.26)	< 0.001	0.89 (0.83–0.96)	0.002	1.18 (0.95–1.47)	0.139		
Cerebrovascular disease	1.01 (0.93–1.09)	0.889			1.25 (0.95–1.63)	0.112		
CVA	1.07 (0.94–1.20)	0.299	0.83 (0.73–0.94)	0.003	0.94 (0.61–1.44)	0.776		
TIA	1.08 (0.95–1.22)	0.233	1.50 (1.33–1.70)	< 0.001	1.27 (0.84–1.91)	0.256		
Carotid interventions	1.18 (0.90–1.56)	0.238			0.80 (0.26–2.48)	0.697		
Chronic lung disease	1.15 (1.06–1.25)	0.001			1.58 (1.19–2.11)	0.002	1.63 (1.22–2.19)	0.001
Asthma	1.70 (1.51–1.92)	< 0.001			1.48 (0.97–2.27)	0.070		
LVEF	0.65 (0.59–0.70)	< 0.001			1.00 (0.99–1.00)	< 0.001		
CAD								
1 VD	1.02 (0.96–1.09)	0.483			0.95 (0.78–1.14)	0.554		
2 VD	1.11 (1.05–1.17)	< 0.001			1.14 (0.96–1.34)	0.136		
3 VD	0.83 (0.80–0.87)	< 0.001			0.82 (0.70–0.96)	0.012		
LM disease	1.02 (0.97–1.08)	0.387			1.04 (0.86–1.27)	0.664		
Previous MI	2.17 (2.05–2.29)	< 0.001	1.75 (1.65–1.86)	< 0.001	1.37 (1.09–1.73)	0.007	1.33 (1.05–1.68)	0.016
Previous PCI	4.53 (4.20–4.88)	< 0.001	3.61 (3.34–3.89)	< 0.001	2.81 (2.21–3.58)	< 0.001	3.19 (2.49–4.08)	< 0.001
Redo surgery	1.23 (1.17–1.29)	< 0.001			1.73 (0.50–9.15)	0.617		
Cardiogenic shock	1.27 (1.06–1.52)	0.011			1.81 (0.25–12.87)	0.555		
Critical preoperative state	1.65 (1.35–2.02)	0.000			1.54 (0.69–3.45)	0.293		

IABP	1.71 (1.52–1.93)	0.010			1.62 (0.39–9.04)	0.507		
iv. nitrates	1.13 (1.06–1.20)	0.093	1.18 (1.11–1.26)	< 0.001	1.02 (0.81–1.29)	0.845		
iv. inotropes	1.08 (0.94–1.25)	0.271			3.88 (1.60–9.39)	0.003		
Elective	1.07 (1.03–1.11)	0.190			0.97 (0.89–1.07)	0.277		
Urgent	1.29 (1.08–1.54)	0.032			1.67 (0.47–8.16)	0.424		
Emergency	1.67 (1.38–2.04)	0.021			1.67 (0.23–11.84)	0.614		
Salvage	2.02 (1.51–2.73)	0.000			1.37 (0.77–2.81)	0.326		
Surgery								
OPCAB	1.05 (0.99–1.10)	0.083			1.13 (0.97–1.32)	0.113		
CPB-CABG	0.96 (0.92–1.00)	0.080			0.86 (0.73–1.02)	0.079		
MIDCAB	0.90 (0.72–1.13)	0.373			1.31 (0.83–2.07)	0.246		
Aortic no-touch	0.93 (0.82–1.07)	0.226			0.60 (0.09–4.26)	0.611		
Hybrid procedure	0.94 (0.60–1.48)	0.800			0.60 (0.09–4.26)	0.611		
CABG + MV	1.01 (0.96–1.06)	0.789			1.12 (0.89–1.42)	0.581		
CABG+AV	1.02 (0.96–1.09)	0.544			0.92 (0.68–1.24)	0.336		
CABG+TV	0.99 (0.92–1.06)	0.709			0.81 (0.56–1.18)	0.278		
CABG+multiple valves	1.00 (0.94–1.07)	0.681			0.95 (0.71–1.28)	0.398		
CABG+other	1.02 (0.94–1.07)	0.645			1.06 (0.97–1.11)	0.677		
Grafts and anastomoses								
RIMA/LIMA	0.98 (0.93–1.03)	0.488			1.06 (0.89–1.26)	0.521		
BIMA	0.92 (0.79–1.07)	0.287			0.95 (0.54–1.66)	0.855		
Pedicled IMA	1.00 (0.96–1.05)	0.081			1.00 (0.95–1.05)	0.501		
Skeletonized IMA	0.93 (0.82–1.07)	0.226			0.93 (0.80–1.09)	0.300		
Radial artery	0.92 (0.77–1.10)	0.300			1.02 (0.97–1.07)	0.094		
Arterial anastomoses	0.94 (0.71–1.27)	0.513			0.94 (0.83–1.08)	0.239		
Venous anastomoses	0.98 (0.78–1.27)	0.794			0.93 (0.78–1.11)	0.313		
Sequential anastomoses	1.01 (0.96–1.07)	0.666			0.95 (0.72–1.29)	0.526		
Composite anastomoses	1.00 (0.94–1.07)	0.627			0.99 (0.79–1.28)	0.807		
Total arterial revascularization	1.00 (0.93–1.07)	0.695			1.03 (0.97–1.09)	0.679		
Completeness of revascularization	1.00 (0.95–1.06)	0.876			1.12 (0.91–1.37)	0.283		
Late survival	0.83 (0.73–0.94)	0.005			0.74 (0.56–0.98)	0.036	0.42 (0.15–0.65)	< 0.001

PS: propensity score; CVA: cerebrovascular accident; TIA: transient ischemic attack; LVEF: left ventricle ejection fraction; CAD: coronary artery disease; VD: vessel disease; MI: myocardial infarction; PCI: percutaneous coronary intervention; IABP: intra-aortic balloon pump; iv: intravenous; OPCAB: Off-Pump Coronary Artery Bypass; CPB: cardiopulmonary bypass; MIDCAB: Minimally Invasive Direct Coronary Artery Bypass; CABG: coronary artery bypass grafting; MV: mitral valve; AV: aortic valve; TV: tricuspid valve; LIMA/RIMA/BIMA: Left/Right/Bilateral Internal Mammary Artery.

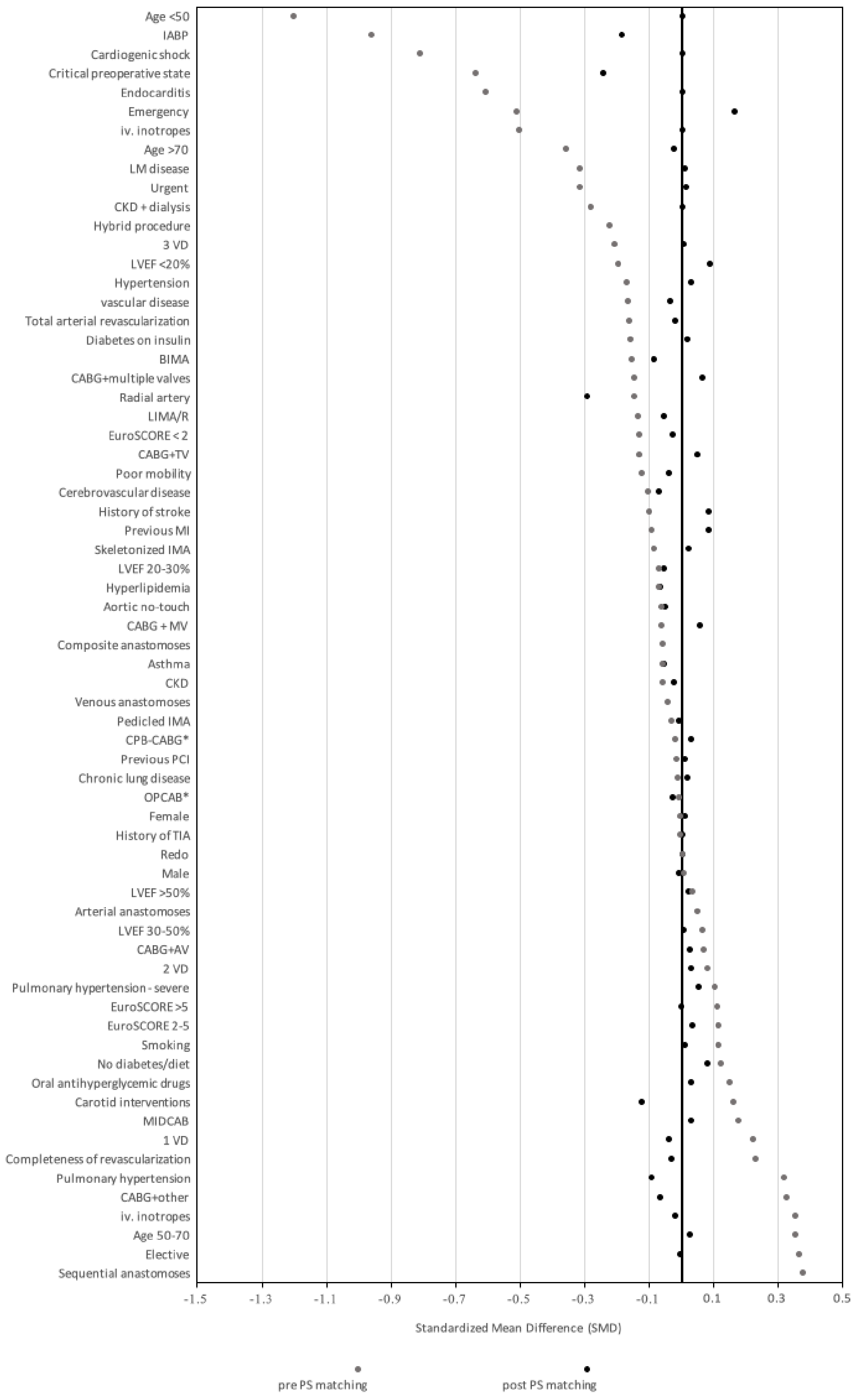

Figure S1. Standardized differences before and after propensity score matching comparing covariate values for patients undergoing concomitant surgical ablation vs. no concomitant surgical ablation.
